# Structural Characterization and Protective Effects of CPAP-1, an Arabinogalactan from *Curcuma phaeocaulis* Val., Against H_2_O_2_-Induced Oxidative Damage in HUVECs

**DOI:** 10.3390/molecules30224340

**Published:** 2025-11-09

**Authors:** Yuhao Long, Sirui Yi, Huizhi Zhou, Fangrou Chen, Yiping Guo, Li Guo

**Affiliations:** 1 Key Laboratory of Standardization of Chinese Medicine, Chengdu University of Traditional Chinese Medicine, Ministry of Education, Chengdu 611137, China; longyuhao@stu.cdutcm.edu.cn (Y.L.); yisirui@stu.cdutcm.edu.cn (S.Y.); 2 School of Pharmacy, Chengdu University of Traditional Chinese Medicine, Chengdu 611137, China; zhouhuizhi@stu.cdutcm.edu.cn; 3 College of Clinical Medicine, Chengdu University of Traditional Chinese Medicine, Chengdu 611137, China; chenfangrou@stu.cdutcm.edu.cn

**Keywords:** *Curcuma phaeocaulis*, isolation and purification, structural characterization, oxidative stress

## Abstract

*Curcuma phaeocaulis*, a perennial herb of the ginger family, has been used to treat many diseases in traditional medicine systems. This study aimed to extract, isolate, and purify a homogeneous polysaccharide from *C. phaeocaulis*, conduct preliminary structural characterization, and evaluate its antioxidant activity at the cellular level. The structure of the purified polysaccharide (CPAP-1) was characterized using size exclusion chromatography (SEC), chemical derivatization analysis (CDA), GC-MS, FT-IR, and NMR. The results showed that CPAP-1 has an apparent molecular weight of 118.122 kDa and is hypothesized to be an arabinogalactan with a backbone composed of →3,6)-*β*-d-Gal*p*-(1→ and →3)-*β*-d-Gal*p*-(1→ residues, a structure that is relatively novel in *Curcuma longa*. In vitro antioxidant assays demonstrated that CPAP-1 possesses potent antioxidative stress activity, effectively scavenging both DPPH and hydroxyl radicals. Furthermore, cellular experiments revealed that at concentrations of 500 and 750 mg/L, CPAP-1 significantly protected human umbilical vein endothelial cells (HUVECs) against H_2_O_2_-induced oxidative damage. In conclusion, these findings suggest that CPAP-1 could be developed as a natural antioxidant, functional food, or therapeutic agent for preventing and mitigating oxidative stress-related vascular injury, providing a theoretical basis for further development and application.

## 1. Introduction

Chinese medicinal extracts prominently feature polysaccharides, proteins, and peptides as vital macromolecules. Their significance as primary bioactive constituents underpinning therapeutic efficacy has driven substantial scientific interest in the past few years. Polysaccharides have emerged as a key focus in contemporary studies on traditional Chinese medicine, thanks to their complex molecular structures and impressive therapeutic properties. These naturally occurring compounds show exceptional promise in regulating immune responses [1], combating oxidative stress [2], and inhibiting tumor growth [3], making them particularly valuable in pharmacological research.

*Curcuma longa*, belonging to the ginger family, is a prominent botanical source in traditional Chinese medicine. It has been widely prescribed in clinical practice to invigorate the stomach, eliminate stomachache, dissolve blood stasis, and relieve pain. The polysaccharides derived from *Curcuma longa* serve as bioactive components, exhibiting antitumor properties [4], promoting wound healing [5], and enhancing immune functions [6]. As a member of the *Curcuma longa* family, *Curcuma phaeocaulis* Val. is rich in polysaccharides and exhibits various pharmacological activities. It has been used as a traditional herbal remedy to control disorders associated with stasis, gastritis, and pain [7]. Phytochemical studies have identified various bioactive constituents in *C. phaeocaulis*, especially the essential oil and its constituent sesquiterpenes, which exhibit a range of therapeutic properties against cancer [8], inflammation [9], and oxidative stress [10].

However, investigations into the structural characterization and pharmacological properties of polysaccharides in *C. phaeocaulis* are limited. To address this gap, this study systematically isolated and purified an arabinogalactan for structure analysis. Monosaccharide composition analysis indicated galactose as the most abundant component. Considering that galactose typically adopts the stable pyranose form, which is conformationally favorable for the polysaccharide backbone formation, the polysaccharide might feature a backbone of *β*-galactopyranosyl residues with arabinose and xylose constituting the side chains. Subsequent structural characterization was performed to verify this hypothesis by elucidating the specific composition and linkage patterns. The compound’s antioxidant properties were then assessed through DPPH and hydroxyl radical scavenging assays. Furthermore, initial investigations were conducted to examine its potential in mitigating H_2_O_2_-induced oxidative damage in HUVEC cells.

## 2. Results

### 2.1. Extraction and Isolation of CPAP-1

To extract the crude polysaccharide CPP, the dried *C. phaeocaulis* powder underwent a series of processes. Initially, the powder was subjected to a water extraction and alcohol precipitation technique. Then, the precipitate was redissolved in distilled water. Next, the solution was deproteinized and decolorized, followed by dialysis and, finally, lyophilization to yield the desired CPP. Following separation via an anion-exchange column (DEAE seplife FF), a neutral (CPNP) and an acidic (CPAP) polysaccharide were isolated. The anion-exchange chromatographic profile of CPP was obtained using a DEAE seplife FF column (Figure S1). CPAP components, after testing, were found to possess high purity; therefore, further purification processing was conducted. Size exclusion chromatography was employed through a Sephacryl S-400 HR column (General Electric Company, Cincinnati, OH, USA). The elution profile is depicted in Figure S2. This ultimately yielded the purified polysaccharide CPAP-1, which presented as a colorless powder and dissolved readily in water. The extraction rates and purities of each polysaccharide component are displayed in Table S1.

**Figure 1:**
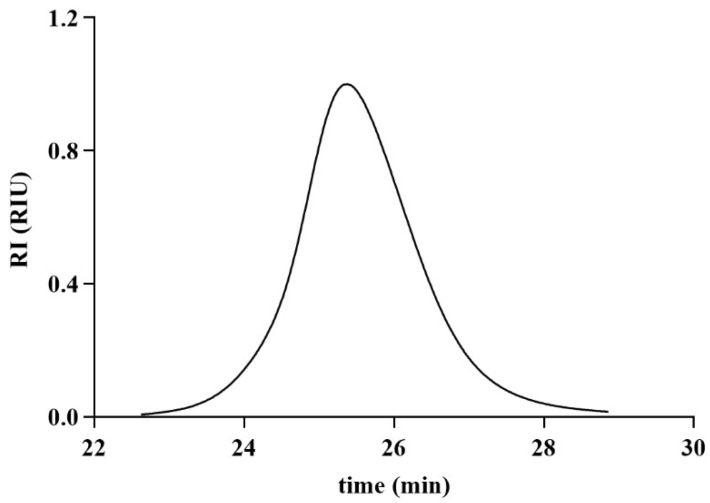
Chromatography profile of CPAP-1 by SEC.

The SEC chromatogram of CPAP-1 revealed a single, symmetrical peak (Figure 1). Calibration with a dextran standard curve indicated a molecular weight of 118.122 kDa.

### 2.2. Structural Characterization of CPAP-1

#### 2.2.1. Monosaccharide Composition Results

**Figure 2:**
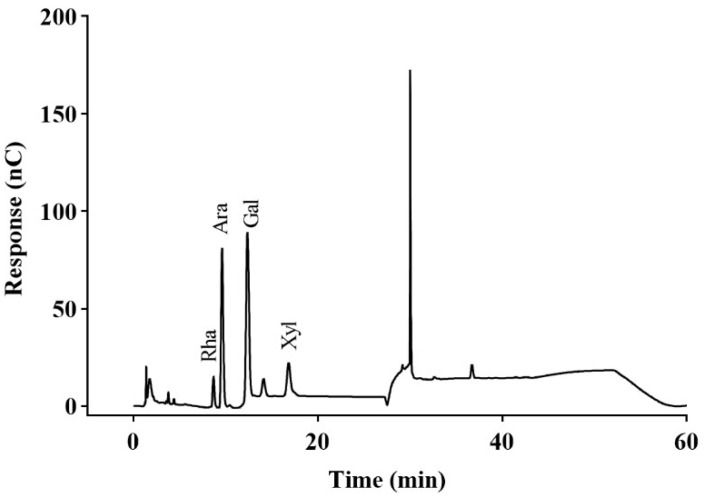
Ion chromatogram of CPAP-1.

The monosaccharide composition analysis was performed via hydrolysis with 2 mol/L TFA at 120 °C, and the results are presented in Figure 2. CPAP-1 was primarily made up of galactose (Gal, 38.65%), arabinose (Ara, 33.05%), and xylose (Xyl, 10.89%), with just a smidge of rhamnose (Rha, 6.37%).

#### 2.2.2. Infrared and Ultraviolet Scanning Results

**Figure 3:**
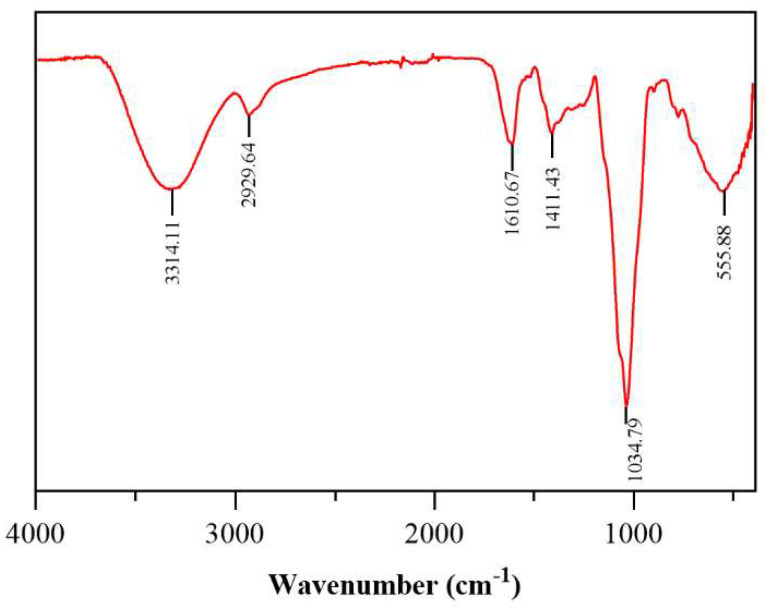
Infrared spectrogram of CPAP-1.

**Figure 4:**
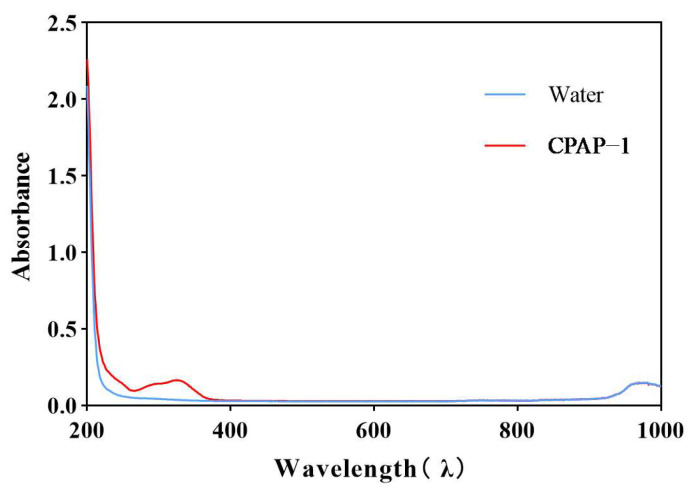
Ultraviolet spectrogram of CPAP-1.

The IR spectrum of CPAP-1 (Figure 3) showed a distinct 3600–3200 cm^−1^ absorption band, indicative of polysaccharide O-H stretching. A strong absorption peak at 3314.11 cm^−1^ suggested O-H stretching, a key polysaccharide marker. FT-IR analysis further revealed key absorption bands at 2929.64 cm^−1^ (C-H stretch) and 1034.79 cm^−1^ (C-O stretch). The results of UV scanning are shown in Figure 4. The absorbance of CPAP-1 at 280 nm was only 0.117, indicating that it contains a trace amount of protein.

#### 2.2.3. Methylation Results

The methylation analysis result (Table 1) suggests that the polysaccharide branched chains are likely capped with a high percentage of t-Ara(*f*). Furthermore, it appears that 3,6-Gal(*p*) and 3-Gal(*p*), found in higher concentrations, probably constitute the backbone of the main chain. Branch structures such as t-Rha(*p*), t-Gal(*p*), 3-Ara(*f*), 4-Xyl(*p*), 4-Glc(*p*)-UA, and 2,3,5-Ara(*f*) were also present. These results, combined with the 2D NMR analysis, enabled the structural inference of the polysaccharide.

#### 2.2.4. NMR Results

**Figure 5:**
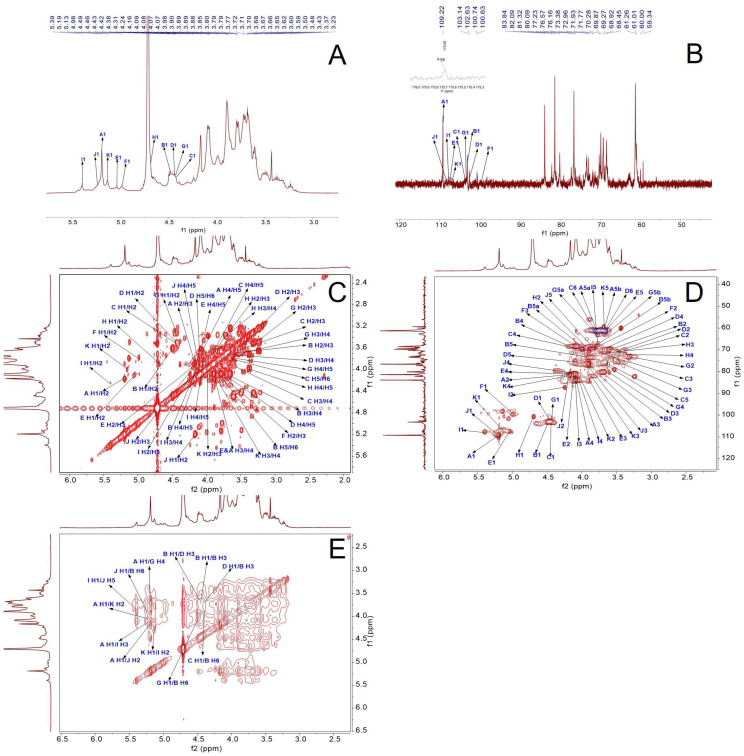
NMR spectra of the CPAP-1. (**A**–**E**): ^1^H NMR, ^13^C NMR, COSY, HSQC, and NOESY spectra. The blue arrows indicate the signal assignments for C and H atoms of each residue.

The backbone of CPAP-1 was deduced via NMR spectroscopy. Based on correlations in HSQC and HMBC spectra, we assigned the ^1^H- and ^13^C-NMR signals and referenced previously reported data for signal assignment. ^1^H NMR signals (Figure 5A) were predominantly observed within the *δ*_H_ range of 3.0 to 5.5 ppm. Multiple linkages were identified within the anomeric zone (*δ*_H_ 4.4–5.5 ppm). This indicated the polysaccharide contained multiple sugar residues. GC-MS corroborated these findings. The most distinguishing anomeric proton peaks exhibited chemical shifts at *δ*_H_ values of 4.41, 4.43, 4.44, 4.48, 4.69, 4.98, 5.03, 5.13, 5.19, 5.24, and 5.39. Likewise, unique anomeric carbon resonances appeared in the ^13^C NMR profile (Figure 5B). Their chemical shifts were *δ*_C_ 99.55, 100.68, 102.63, 103.14, 103.31, 103.57, 106.98, 107.47, 107.74, 108.42, and 109.22. Moreover, anomeric proton and carbon resonances were assigned within the sample. The anomeric signals were pinpointed by correlating cross-peaks across three spectra: ^1^H NMR, ^13^C NMR, and HSQC (Figure 5D). The chemical shifts (*δ*_H/C_, ppm) for these signals were identified as follows: 5.19/109.22, 4.48/103.14, 4.41/103.57, 4.44/102.63, 5.03/107.47, 4.98/99.55, 4.43/103.31, 4.69/100.68, 5.39/107.74, 5.24/108.42, and 5.13/106.98. This analysis leveraged overlapping spectral data to confirm the anomeric region assignments. They were noted and, respectively, named Residue A through K.

Based on HSQC spectral analysis and the published literature [11,12,13], the cross-peaks with stronger signals *δ*_H/C_ 5.19/109.22, 4.48/103.14, and 4.41/103.57 were attributed to *α*-l-Ara*f*-(1→, →3,6)-*β*-d-Gal*p*-(1→, and *β*-d-Gal*p*-(1→ of H-1/C-1; other related signals 4.44/102.63, 5.03/107.47, 4.98/99.55, 4.43/103.31, 4.69/100.68, 5.39/107.74, 5.24/108.42, and 5.13/106.98 were attributed to →3)-*β*-d-Gal*p*-(1→, →3)-*α*-l-Ara*f*-(1→, →4)-*α*-d-Glc*p*A-(1→, →4)-*β*-d-Xyl*p*-(1→, *β*-l-Rha*p*-(1→, →2,3,5)-*α*-l-Ara*f*-(1→, →2,5)-*α*-l-Ara*f*-(1→, →2)-*α*-l-Ara*f*-(1→, →2)-*α*-l-Ara*f*-(1→ of H-1/C-1 [13,14,15,16,17,18,19,20]. The positioning of sugar molecules was pinpointed by analyzing the specific shifts in their anomeric chemistry. In the case of pyranose structures, a proton signal at over 4.8 ppm on the NMR spectrum signaled an *α*-configuration, while anything below that mark denoted a *β*-configuration [21]. The anomeric protons in *α*-configured furanose units exhibited chemical shifts spanning 4.9 to 5.4 ppm, while their corresponding carbon resonances fell within the 105–110 ppm range. This spectral data indicated that Residues A, E, F, I, J, and K adopt the *α*-configuration, whereas Residues B, C, D, G, and H exist in the *β*-configuration. Furthermore, the H-2 proton signal was pinpointed through its correlation with the anomeric proton’s chemical shift, with their coupling interaction visibly demonstrated by the H-1/H-2 cross-peak in ^1^H-^1^H COSY (Figure 5C). Similarly, chemical shifts for other protons in each sugar residue were successively identified. The HSQC 2D spectrum provided the necessary data to map out the crucial chemical shifts for C-2, C-3, C-4, C-5, and C-6 in the sugar moieties. As shown in Table 2, these values correspond to the monosaccharide residues present in CPAP-1.

**Figure 6:**
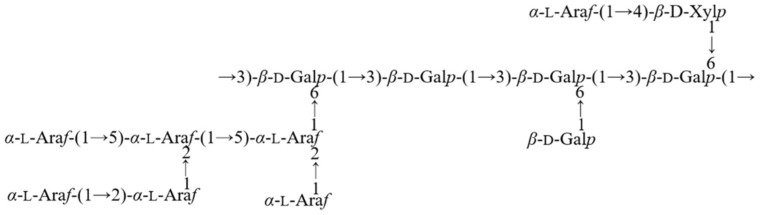
Structure of the putative CPAP-1 glycan chain.

HMBC and NOESY spectral analysis (Figure 5E) revealed CPAP-1 polysaccharide linkage sites and sugar residue sequences. The HMBC spectra revealed no discernible signals corresponding to these linkages. NOESY data revealed a *δ*_H/H_ 5.19/3.68 cross-peak (A H-1/G H-4), confirming Residue A’s *O*-1 linkage to Residue G’s C-4. Other relevant signals *δ*_H/H_ 5.19/4.13 (A H-1/I H-3), 4.48/3.70 (B H-1/B H-3), 5.19/4.17 (A H-1/J H-2), 5.19/4.01 (A H-1/K H-2), and 4.48/3.61 (B H-1/D H-3) indicate that Residue A’s *O*-1 links up with Residue I’s C-3, and the *O*-1 of Residue B is tethered to the C-3 on another Residue B molecule. Residue A forms a bond between its *O*-1 position and the C-2 carbon of both Residue J and Residue K. Meanwhile, Residue B’s *O*-1 is linked to the C-3 atom of Residue D. The signals 4.41/3.87 (C H-1/B H-6) and 4.41/3.99 (C H-1/B H-6) confirmed the linkage between Residue C *O*-1 and Residue B C-6. Correlation signals *δ*_H/H_ 4.44/3.70 (D H-1/B H-3), 4.43/3.87 (G H-1/B H-6), 4.43/3.99 (G H-1/B H-6), 5.39/3.85 (I H-1/J H-5), 5.24/3.87 (J H-1/B H-6), and 5.13/4.24 (K H-1/I H-2) indicate that the *O*-1 linkage of Residue D hooks up with the C-3 spot on Residue B, while the *O*-1 on Residue G links to the C-6 region of Residue B. This pattern continues with Residue I, whose *O*-1 is also tethered to the C-6 section of Residue B. Additionally, the *O*-1 of Residue J forms a bond with the C-5 site on itself, and another bond connects the *O*-1 on Residue J to the C-6 of Residue B. Finally, the *O*-1 of Residue K is joined to the C-2 location on Residue I; Residue E, F, and H failed to participate in the linkage of the sugar chain due to their weak signals in their profiles. By merging the data from the NMR and GC-MS analyses, it became clear that CPAP-1 predominantly links up with both →3,6)-*β*-d-Gal*p*-(1→ and →3)-*β*-d-Gal*p*-(1→ to create the primary backbone. Additionally, the side branches are largely made up of *α*-l-Ara*f*-(1→4)-*β*-d-Xyl*p*-(1→, which attach to the *O*-6 position of the →3,6)-*β*-d-Gal*p*-(1→, while *β*-d-Xyl*p*-(1→ also connects to this position at *O*-6. A proposed model for the structure of the CPAP-1 glycan chain is shown in Figure 6.

#### 2.2.5. Electron Microscope Scanning Results

**Figure 7:**
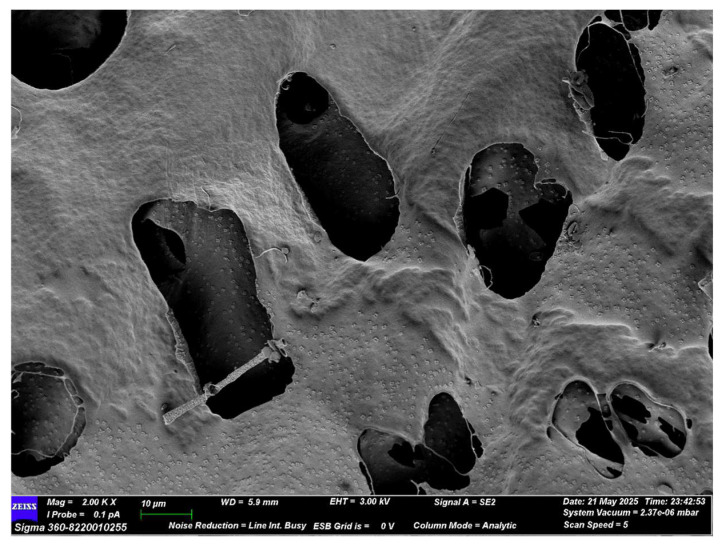
CPAP-1 electron microscope scan (×2000).

Figure 7 shows that CPAP-1 primarily displays a flaky morphology with holes. Its surface is generally smooth and shows a certain degree of network-like structure.

#### 2.2.6. Results of X-Ray Diffraction Measurements

**Figure 8:**
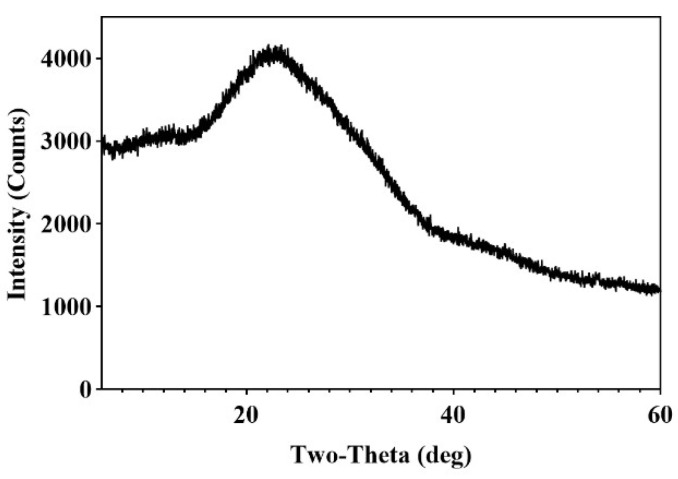
XRD result of the CPAP-1.

Usually, polysaccharides exhibit crystalline structures with distinct, narrow diffraction peaks, whereas amorphous structures are characterized by wider diffraction peaks. Figure 8 shows that the highest peak of CPAP-1 appeared around the 2*θ* of 20°, and there was one broad peak with greater intensity at the 2*θ* of 22.62°, and only a few weak diffraction peaks existed in other ranges, which indicated that CPAP-1 was an amorphous structure.

### 2.3. Results of Antioxidant Activity Assay

#### 2.3.1. DPPH Radical Scavenging Activity

**Figure 9:**
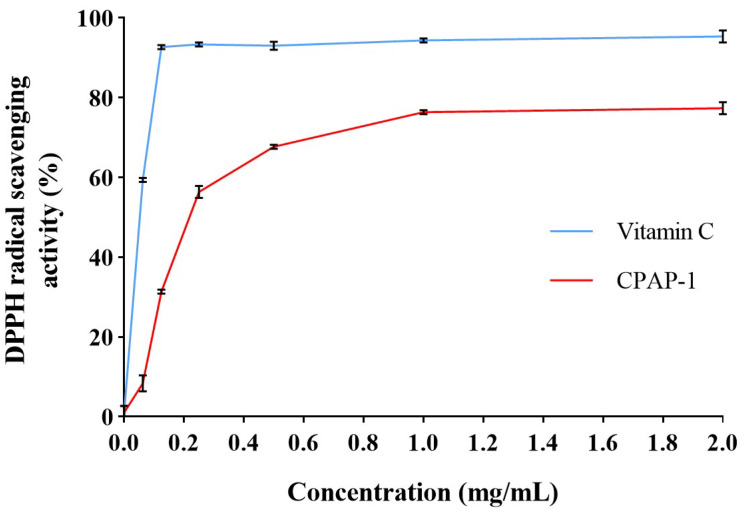
Comparison of DPPH free radical scavenging effects of CPAP-1 and vitamin C.

The DPPH assay was used as an initial screening mechanism to ascertain the polysaccharides’ antioxidant capabilities. This widely recognized free radical compound provided insights into both the reducing agents present in polysaccharides and their ability to neutralize reactive oxygen species. The test measured polysaccharides’ capacity to counteract radicals and alleviate oxidative stress. Figure 9 demonstrates dose-dependent effects for both CPAP-1 and ascorbic acid in this assay. In this assay, Figure 9 shows that both CPAP-1 and vitamin C exhibit dose-dependent behavior. The efficacy of CPAP-1 in scavenging free radicals skyrocketed, leaping from 8.33% to 77.33% as the concentration was bumped up. At a concentration of 1.0000 mg/mL, CPAP-1 demonstrated a 76.33% success rate in neutralizing DPPH free radicals, and this efficiency climbed even higher to 77.33% when the concentration was doubled to 2.0000 mg/mL.

#### 2.3.2. Scavenging of Hydroxyl Radicals

**Figure 10:**
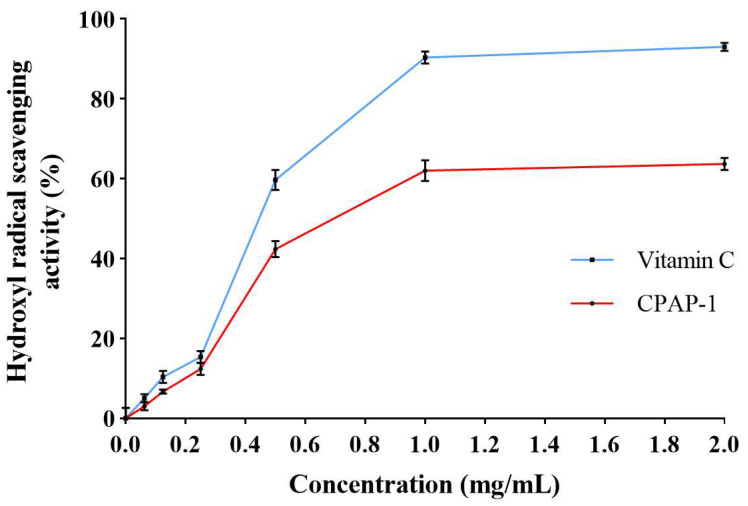
Evaluation of the antioxidant properties of CPAP-1 and vitamin C.

Among antioxidant properties, the hydroxyl radical (OH·) was the most reactive. It caused severe damage to biomolecules. Ascorbic acid and CPAP-1 demonstrated scavenging effects against the hydroxyl radical. These effects are presented in Figure 10. CPAP-1 demonstrated a dose-dependent ability to neutralize hydroxyl radicals, with its scavenging activity ranging between 0.0625 and 2.0000 mg/mL. As the concentration rose, its effectiveness climbed sharply from 3.00% to 63.67% inhibition rate. CPAP-1 exhibited hydroxyl radical scavenging at 62.00% (1.0000 mg/mL) and 63.67% (2.0000 mg/mL).

### 2.4. Impact of CPAP-1 on H_2_O_2_-Injured HUVEC Proliferation

**Figure 11:**
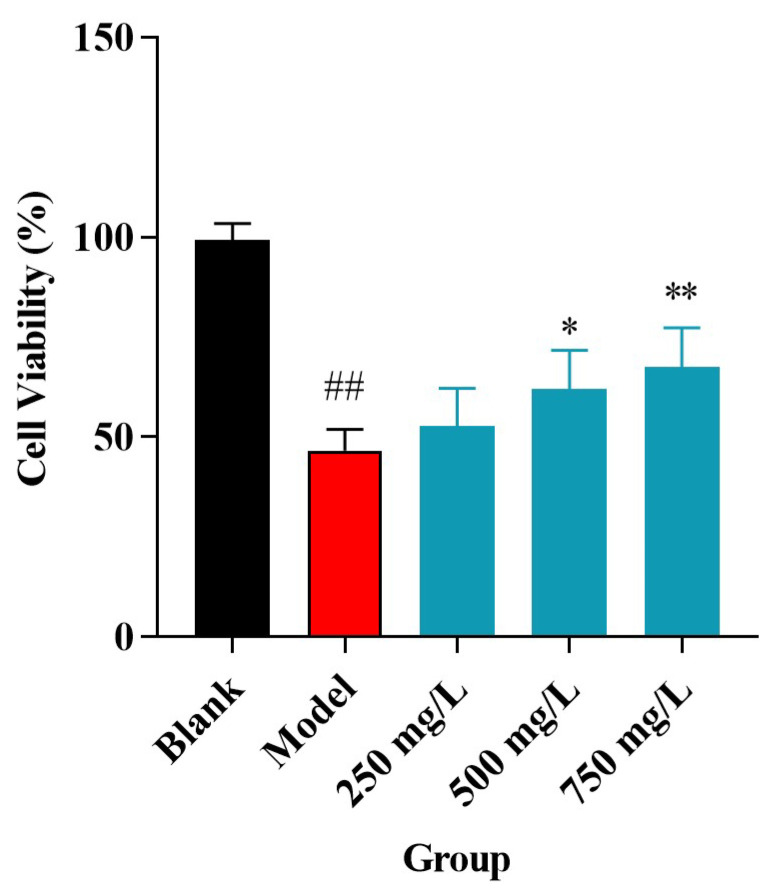
Impact of CPAP-1 on the proliferation of H_2_O_2_-injured HUVEC cells. The values represent the means ± SD of triplicate trials. ^##^ *p* = 0.00004 compared to the blank group; * *p* = 0.0410 and ** *p* = 0.0096 compared to the H_2_O_2_-injured group.

As shown in Figure 11, H_2_O_2_ treatment significantly reduced HUVEC viability to 46% compared to the blank group (set as 100%). However, co-treatment with CPAP-1 at 500 and 750 mg/L significantly attenuated this injury, increasing cell viability to 61.95% (*p* = 0.0410) and 67.62% (*p* = 0.0096), respectively, compared to the model group. This protective effect on cell viability is consistent with our previous findings on the antioxidant activity of CPAP-1.

## 3. Discussion

A variety of methods are available for the extraction, purification, and isolation of polysaccharides from *Curcuma longa*. Techniques such as hot water extraction, complex enzyme extraction, microwave-assisted extraction, ultrasonic extraction, and dilute alkaline extraction have been employed for this purpose. Hot water extraction is widely used and the most mature due to its simplicity and low cost [22]. In clinical practice, soup is the most common clinical dosage form of traditional Chinese medicine, and the aqueous extract of Chinese medicine obtained by hot water extraction contains a large number of polysaccharide components, which enter the body through oral intake and thus exert therapeutic effects. Therefore, in this experiment, the hot water extraction method was chosen to extract polysaccharides, which were then separated and purified. In this research, crude polysaccharides (CPP) were initially isolated via hot water extraction and subsequent ethanol precipitation. This CPP preparation underwent fractionation using a DEAE seplife FF column [23]. This process yielded two distinct fractions: CPNP and CPAP. A homogeneous polysaccharide (CPAP-1) was separated from the acidic polysaccharide (CPAP) using a Sephacryl S-400 HR column (General Electric Company, Cincinnati, OH, USA) [23]. CPAP-1’s monosaccharide profile indicated a high concentration of galactose, arabinose, and xylose. Scanning electron microscopy further revealed that CPAP-1 displayed a flake-like morphology with surface porosity and an overall smooth surface.

In the theory of traditional Chinese medicine, *C*. *phaeocaulis* has the effect of breaking blood and moving qi, eliminating stagnation and relieving pain, and restoring the smooth flow of blood. Modern research has found that oxidative stress not only triggers atherosclerosis and corresponding cardiovascular diseases through excitation of sympathetic nerves and activation of vascular cell adhesion factors [24] but is also associated with increased risk of aging, diabetes, and neurodegenerative diseases [25]. This implies that there may be some unknown polysaccharide components with antioxidative stress activity in *C*. *phaeocaulis*, which is worth studying. Therefore, the present experiment was carried out to investigate the pharmacology of polysaccharides of *C*. *phaeocaulis* in terms of their antioxidative stress activity. DPPH is simple and reproducible and is often used as a means of preliminary and rapid screening of polysaccharides with antioxidant activity. A polysaccharide that effectively scavenges DPPH free radicals indicates that it is inherently reducing. Therefore, a polysaccharide that can effectively scavenge DPPH can be considered a primary antioxidant. The hydroxyl radical, unlike the DPPH radical, is one of the most reactive and destructive reactive oxygen species known, with extremely high activity and a short life span. The ability to scavenge it indicates that polysaccharides have a very powerful antioxidant potential. Conducting two experiments simultaneously allowed for the assessment of the broad spectrum of polysaccharide antioxidant capacity. If the polysaccharide performs well in both experiments, then it is a very promising antioxidant candidate that deserves more in-depth studies at the cellular and animal levels. In vitro antioxidant assays revealed that CPAP-1 exhibits strong dose-responsive antioxidant activity, effectively neutralizing both DPPH and hydroxyl radicals. When tested at 1.0000 mg/mL concentration, the compound demonstrated impressive radical inhibition, quenching over 76% of DPPH radicals and more than 63% of hydroxyl radicals. These findings highlight CPAP-1’s robust free radical scavenging capacity. The above results indicated that CPAP-1 has antioxidant activity in vitro.

Meanwhile, the vascular endothelium not only serves as a barrier for the exchange of substances between blood and tissues but is also an endocrine metabolic organ with multiple functions [26]. Damage to endothelial structure and function is associated with a variety of clinical diseases, such as atherosclerosis [27], hypertension [28], tumors [29], diabetes [30], and Kawasaki disease [31]. Oxidative stress represented a primary cause of vascular endothelial injury [32]. Researchers commonly use H_2_O_2_ to induce oxidative stress in cells, simulating in vivo oxidative damage [33,34,35]. Therefore, the antioxidative stress activity of CPAP-1 was also explored. Cellular experiments showed that CPAP-1 possessed a protective effect against H_2_O_2_ damage to HUVEC cell growth when the concentration of CPAP-1 was 500 and 750 mg/L, which could increase the cell survival rate to 61.95% (*p* < 0.05) and 67.62% (*p* < 0.01), respectively. These results are also consistent with the anti-inflammatory and cardiovascular protective effects of *C*. *phaeocaulis* in clinical practice.

In addition, a clear structure–activity relationship was observed between the chemical characteristics of the different polysaccharide fractions and their antioxidant activity. This observation aligns with findings from Kou et al. [36], who also isolated multiple polysaccharides from other *Curcuma* species and noted differences in their bioactivities. In our study, CPAP-1 (a homogeneous polysaccharide, 118.122 kDa) and CLP-10 (a heterogeneous fraction) showed similar molecular weight ranges and monosaccharide composition. However, CPAP-1 exhibited significantly stronger antioxidant activity. At 500 mg/L, CPAP-1 increased HUVEC cell survival to 67.62%, while CLP-10 only raised it to about 60%. This suggests that when apparent structural characteristics are similar, polysaccharide homogeneity and fine spatial structure may be critical intrinsic factors influencing biological activity. Furthermore, the heterogeneous fraction CLP-50 (50–100 kDa) showed a stronger protective effect at the same concentration, increasing cell survival to over 80%. Its superior activity may come from two sources. First, its monosaccharide composition contained up to 17.47% uronic acids (GlcA and GalA). This agrees with the report from Wang et al. [37], which states that polysaccharides rich in uronic acids often show enhanced antioxidant capacity. Second, as a heterogeneous mixture, CLP-50 contains multiple structural types of polysaccharides. These may work together, creating a synergistic effect that boosts their overall activity. The heterogeneous fraction CLP-100 (>100 kDa), which has a similar molecular weight to CPAP-1, also performed better than CPAP-1. This further supports the idea that structural complexity can enhance function [38].

In summary, our study indicates that the antioxidant stress activity of polysaccharides is not governed by a single structural parameter. Instead, it is co-determined by chemical composition (such as uronic acid content), advanced structural features, and potential synergistic effects between components.

## 4. Materials and Methods

### 4.1. Materials and Experimental Devices

*C*. *phaeocaulis* rhizomes were obtained from Hehuachi Chinese herbal medicine market, Chengdu, China. DEAE Seplife FF (column volume 26 mm × 400 mm) was procured from Xi’an Lanxiao Science and Technology New Material Co., Ltd. (Xi’an, China). The AKTA purification system (explorer 100) and Sephacryl S-400 HR column (26 mm × 1000 mm) were purchased from General Electric Company (Cincinnati, OH, USA). The Ohpak SB-805 HQ (300 × 8 mm) and Ohpak SB-803 HQ (300 × 8 mm) columns were acquired from Shodex (Tokyo, Japan). Monosaccharide standards, including Fucose (Fuc, Lot F2252), Glucose (Glc, Lot D9434), Mannose (Man, Lot M6020), Arachidonose (Ara, Lot E003256), Galactose (Gal, Lot G5388), Glucuronic Acid (Glc-UA, Lot G5269), Galacturonic Acid (Gal-UA, Lot 73960), Xylose (Xyl, Lot 95729), Fructose (Fru, Lot F0550000), Ribose (Rib, Lot R7500), Rhamnose (Rha, Lot B21172), Mannuronic acid (Man-UA, Lot B25941), and Guluronic acid (Gul-UA, Lot B25921) were purchased from Shanghai Yuanye Ltd. (Shanghai, China). Hydrogen peroxide (H_2_O_2_) was procured from Shanghai Macklin Biochemical Technology Company (Shanghai, China). The DPPH Free Radical Scavenging Ability Kit (DPPHFRS.S-W96) and Hydroxyl Radical Test Kit (OH-W96) were acquired from Shanghai Enzyme-link Biotechnology Co. GIBCO (Grand Island, NY, USA) supplied DMEM, supplemented with 10% FBS (FSP500, Excell). HUVEC cells were purchased from Sichuan Biou-Bio Company (Chengdu, China).

The analysis was carried out using a Thermo ICS 5000+ ion chromatography setup (Thermo Fisher Scientific, Waltham, MA, USA), equipped with a Dionex™ CarboPac™ PA20 column (150 × 3.0 mm, 10 μm) for HPLC detection. A 6890A-5977B gas chromatograph (Agilent, Santa Clara, CA, USA) was utilized in the GC-MS analysis. High-purity helium was used as the carrier gas for chromatography. The NMR instrument (AVANCE NEO 600M) was purchased from Bruker (Billerica, MA, USA). A Zeiss Merlin Compact field emission scanning electron microscope (high-resolution) was acquired from Zeiss (Oberkochen, Germany). The Multiskan GO enzyme marker and Nicolet iS-10 FT-IR spectrometer were acquired from Thermo, and the X-ray diffractometer (Ultima IV) was purchased from Rigaku (Tokyo, Japan).

### 4.2. Polysaccharide Retrieval and Refinement

Following the extraction method of reference [5] with slight modification, *C. phaeocaulis* rhizomes (1 kg, desiccated) were mechanically ground and then sieved (60 mesh) to yield uniform powder. Petroleum ether (material–liquid ratio of 1:5) was added, stirred well, and soaked overnight at ambient temperature to eliminate fat-soluble compounds and some low-molecular-weight impurities. This process facilitated preliminary purification. The next day, the petroleum ether was centrifuged and discarded, leaving the powdered herbs to dry naturally. It was extracted in a 60 °C water bath for 4 h using a 1:20 material-to-water ratio, followed by centrifugation at 6000× *g* for 10 min. The supernatant extract was collected. The formed sediment was re-extracted under identical parameters and procedures. Then, the two extracts were combined. The extracts were reduced to 10% of their original volume via vacuum distillation. Then, four volumes of anhydrous ethanol were introduced, with subsequent precipitation occurring overnight under low temperature. The solid was separated through centrifugation at 8000× *g* for 10 min, following which it was dried to produce the crude polysaccharide extract. The crude polysaccharide extract was dissolved in 1 L of purified water for full solubilization, followed by overnight digestion with 0.5 g each of papain and *α*-amylase. The enzyme became fully inactive after 10 min of boiling water bath treatment. To the aqueous phase, a 25% trichloromethane aliquot in n-butanol (3:1, *v*/*v*) was incorporated, followed by homogenization. The upper aqueous phase was kept after stratification, the above steps were repeated several times until the lower protein layer disappeared, and the aqueous phase was concentrated by vacuum evaporation to remove the residual organic phase. The recovered aqueous layer’s pH was brought to roughly 8.0 via ammonia introduction, followed by slowly adding 1/4 volume of 30% H_2_O_2_ to remove color. The recovered solution underwent 48 h dialysis (3.5 kDa) to eliminate small-molecule contaminants. Subsequently, centrifugation was performed to eliminate insoluble particulates. Finally, the polysaccharide solution was frozen at −80 °C overnight, followed by lyophilization to yield the crude polysaccharide powder CPP (6.4 g).

The crude polysaccharide CPP (5.0 g) was initially dispersed in 200 mL of deionized water and centrifuged at 10,000× *g* for 10 min, and the supernatant was loaded onto a DEAE seplife FF column pre-installed in the AKTA purification system. The solvent flowed consistently at a steady rate of 4 mL/min. A stepwise elution gradient was implemented, progressing from distilled water to sodium chloride solutions of increasing concentration (0.1 M to 0.3 M), and samples of the effluent were collected at 15-milliliter intervals throughout the process. Each fraction’s absorbance at 490 nm was determined via the phenol–sulfuric assay, allowing for the construction of an ion purification elution curve. The same elution peaks yielded identical fractions, which were then combined for further examination. We reduced the initial solution volume to a quarter using rotary evaporation, and after that, we desalinated it via a 3.5 kDa dialysis. We collected CPNP (1000 mg) and CPAP (800 mg).

The higher-purity CPAP fraction (800 mg) was fractionated on a Sephacryl S-400 HR column that was connected to the AKTA purification system. The sample underwent centrifugation at 10,000× *g* for a duration of 10 min, following purification through gel chromatography to separate fractions. The flow rate was 1 mL/min, with purified water eluting 1.5 column volumes; fractions were collected at 10 mL intervals. Absorbance at 490 nm, quantified via the phenol–sulfuric assay for each fraction, constituted the gel purification elution profile. CPAP-1 (82 mg) was collected.

### 4.3. Purity Assessment and Molecular Weight Approximation

Samples were solubilized in 0.1 M NaNO_3_ (with 0.02% NaN_3_, *w*/*w*) to achieve a concentration of 1 mg/mL and then filtered using 0.45 μm membranes before assay. Two gel exclusion chromatographic columns, Ohpak SB-805 HQ (300 × 8 mm) and Ohpak SB-803 HQ (300 × 8 mm), were used in series. The column was maintained at 45 °C with a 100 μL injection volume. A solution of 0.02% NaN_3_ with 0.1 mol/L NaNO_3_ was used as the mobile phase at a flow rate of 0.6 mL/min in isocratic mode for 75 min. Subsequently, the concentration information was detected by using a detector based on their refractive intensities, and the light scattering information of the macromolecules was detected by using a multiangle laser light scattering instrument with the Mark–Houwink equation to calculate the corresponding molecular weight of each component [39,40].

### 4.4. Monosaccharide Composition

According to the references [41,42], a clean chromatographic vial was used, and a certain mass of polysaccharide powder was weighed. Hydrolysis was achieved by treating the sample with 2 mol/L TFA (1 mL) at a temperature of 121 °C for a duration of 2 h. Nitrogen venting and drying were then applied. Chromatographic methanol cleaning followed by desiccation and subsequent methanol washes (2–3 repetitions) eliminated impurities. Subsequently, the purified sample was reconstituted in deionized water and then loaded into a chromatography vial for testing. A Thermo ICS-5000+ ion chromatography (Thermo Fisher Scientific, Waltham, MA, USA) setup with integrated electrochemical detection was employed to analyze the monosaccharide profile. Chromatographic resolution occurred via a Dionex™ CarboPac™ PA20 (150 × 3.0 mm, 10 μm) column; 5 μL was flushed at a rate of 0.5 mL/min, with the column maintained at 30 °C. The elution gradient was programmed as follows: Starting with a mobile phase composition of 95:5:0 (A/B/C, *v*/*v*/*v*) at 0 min, the ratio shifted to 85:5:10 by the 26 min mark. This mixture was maintained at a steady state until 42 min, when an abrupt change to 60:0:40 occurred at 42.1 min. The gradient then transitioned to 60:40:0 at the 52 min point before snapping back to the initial 95:5:0 ratio at 52.1 min, which was maintained through the 60 min endpoint.

The quantification was performed by the external standard method, and the standard curve was obtained by preparing different concentrations of the control solution and fitting it with Chromeleon 7 software. The standard concentration was graphed on the *x*-axis, and the corresponding peak area on the *y*-axis, across varying standard solution concentrations. This approach established the linear correlation between analyte concentrations and their chromatographic responses, and then calculated the amount of the corresponding compounds in the unknown compound based on the peak areas.

### 4.5. Infrared Scanning Measurement

A trace amount of polysaccharide powder was combined with 200 mg KBr and then processed per the cited methods [43]. The powdered blend was compressed into a thin disc measuring 1 mm in thickness. This compacted sample was then analyzed via Fourier-transform infrared spectroscopy (FT-IR) on a Nicolet iZ-10 spectrometer. The instrument captured spectral readings spanning 4000 to 450 cm^−1^, with 32-scan averaging to improve signal quality.

### 4.6. Ultraviolet Scanning Measurement

A 5 mg/mL polysaccharide aqueous preparation was formulated by accurately measuring a sample and dissolving it in ultra-pure water, as cited in the reference [44]. The solution was analyzed quantitatively using a Thermo Fisher (USA) multifunctional enzyme labeling instrument, with the enzyme labeling plate from Corning (Corning, NY, USA). The scanning started at 200 nm, ended at 1000 nm, and was performed at 1 nm intervals. Ultra-pure water provided the negative control; procedures mirrored sample processing.

### 4.7. Methylation Analysis

A total of 5 mg of CPAP-1 powder was weighed for methylation analysis according to reference [42]. First, the sample was pre-treated. Ultra-pure water (1 mL) was used for dissolution, and then 1 mL of a 100 mg/mL CMC solution was added. The subsequent reaction lasted for 2 h. Following this step, 1 mL of a 2 mol/L imidazole solution was introduced, and the mixture was then split evenly into two separate samples. To the first aliquot, 1 mL of a 30 mg/mL sodium borohydride solution was introduced, while the second aliquot received 1 mL of a 30 mg/mL sodium borodeuteride solution. The reactions continued for three hours. The process was quenched with the addition of 100 μL of glacial acetic acid. Following 48 h of dialysis, the sample was lyophilized. The sample underwent subsequent processing. The specimen was submerged in 500 μL of DMSO, followed by the addition of 1 mg of sodium hydroxide. The concoction was left to sit at room temperature for half an hour before we topped it off with 50 μL of iodomethane. The chemical dance went on for a solid hour. Following that, we stirred in 2 mL of dichloromethane and 1 mL of H_2_O. The mixture was vortex-mixed thoroughly. After spinning the sample down at 8000× *g* for 20 min, the top aqueous layer was carefully pipetted off. After discarding the top aqueous layer with care, the washing step was carried out three more times to guarantee thorough purification. Finally, the bottom layer was collected, the one rich in dichloromethane, for the next stage of the analysis. The solvent was volatilized with a soft nitrogen flow. Subsequently, 100 μL of 2 M TFA was introduced, and the mixture was then subjected to a reaction at 121 °C for a duration of 90 min. The hydrolysate was evaporated at 30 °C under reduced pressure to remove residual solvent. Ammonium hydroxide (2 mol/L, 50 μL) was added, followed by the addition of sodium borodeuteride (1 mol/L, 50 μL). The mixture was fully combined and reacted at 25 °C for 150 min. To quench the reaction, 20 μL of acetic acid was introduced, after which the mixture was dried by evaporating the solvent under nitrogen gas. The residue was rinsed twice using 250 μL of methanol before being dried once more under nitrogen gas. Next, 250 μL of acetic anhydride was introduced, and the solution was vortexed vigorously. The reaction proceeded at 100 °C for 150 min, and then 1 mL of water was added. The mixture was permitted to sit for 10 min. Subsequently, 500 μL of dichloromethane was incorporated into the mixture. The mixture was vortex-mixed thoroughly. The mixture was centrifuged at 8000× *g* for 10 min. The aqueous layer was removed; the wash was thrice-repeated. The dichloromethane fraction was retained for GC-MS analysis.

Chromatographic analyses employed an Agilent 6890A GC system (Agilent, USA) featuring a BPX70 capillary column (30 m × 0.25 mm × 0.25 μm). A tiny, 1 μL sample was injected, splitting it 10:1, and ultra-pure helium was used to carry it through the system. Initially, the column was kept at a steady 140 °C for two minutes. After that, the temperature was cranked up to 230 °C at a rate of 3 °C per minute and held there for a final three minutes. The mass spectrometry analysis was conducted on an Agilent 5977B quadrupole MS system (Agilent, USA), featuring an electron ionization source and operated via MassHunter workstation software (B.08.00). Samples were analyzed in full scan mode using electron impact ionization, with the mass spectrometer scanning across a range of 50 to 350 *m*/*z*.

### 4.8. NMR Analysis

According to reference [45], precisely measured CPAP-1 was solubilized in D_2_O, yielding a 40 mg/mL solution, which was lyophilized twice to ensure full removal of H_2_O. The lyophilized material was dissolved again in D_2_O. The prepared solution was cautiously loaded into an NMR tube. A volume of 0.5 mL was used, and the sample in an NMR tube was loaded into a Bruker 600 MHz spectrometer for analysis. Precise quantification of the compound was carried out, with the probe temperature maintained at 25 °C. The instrument acquired comprehensive spectral data, including ^1^H and ^13^C one-dimensional spectra along with a suite of two-dimensional experiments—COSY, HSQC, NOESY, and HMBC—to fully characterize the molecular structure.

The target compound was characterized using a 600 MHz NMR spectrometer (Bruker, Berlin, Germany) at 25 °C. Measurements were conducted with a QXI ^1^H/^31^P/^13^C/^15^N 5 mm quad-resonance inverse detection probe (Z-gradient, ATM Acc), which delivered a ^1^H signal-to-noise ratio of 888 and a resolution of 0.32 Hz (rotating). Additionally, a BBFO ^1^H-^19^F, ^31^P-^15^N forward detection probe (Z-gradient, ATM) was employed, achieving a ^1^H signal-to-noise ratio of 798 with a resolution of 0.26 Hz (rotating), along with a ^13^C signal-to-noise ratio of 328 and a resolution of 0.1 Hz.

### 4.9. Electron Microscope Scanning

A minimal quantity of CPAP-1 was affixed to conductive carbon tape for subsequent analysis according to reference [46] and sprayed with gold to ensure its conductivity. Microstructural images were acquired using a Zeiss Merlin FE-SEM operated at high resolution. The magnification ranged from 500× to 10,000×.

### 4.10. X-Ray Diffraction Measurement

The CPAP-1 sample (20 mg) was weighed on a platform following reference [47], compacted, and then evenly dispersed before being subjected to an X-ray diffraction analysis. This analysis was conducted using a copper target (Cu-Kα radiation, *λ* = 0.15406 nm) operating at 1600 W (40 kV, 40 mA). Data collection was performed with an Ultima IV diffractometer (185 mm radius), scanning a 2*θ* range from 5° to 60° at a rate of 2° per minute, with a step size of 0.02°. The divergence slit was 1/2° and the horizontal slit of the divergence was 10 mm; the anti-scattering divergence slit was 1/2°, the divergence horizontal slit was 10 mm, and the anti-scattering slit was 8.0 mm.

### 4.11. Antioxidant Activity Assay

In the in vitro antioxidant assay, vitamin C served as a positive control for antioxidant activity in this study, and the sample concentrations were 0.0625, 0.1250, 0.2500, 0.5000, and 1.0000 mg/mL, each tested three times in parallel.

#### 4.11.1. DPPH Free Radical Scavenging Activity

Following the procedure outlined in reference [48], with minor adjustments, a 50 μL aliquot of each sample solution (at varying concentrations) was mixed with 200 μL of 0.004% DPPH solution (prepared in anhydrous methanol) in a 96-well plate. The reaction was carried out at 37 °C for one hour under light-protected conditions. The concoction was spun down at 12,000× *g* for a quick 5 min, and the absorbance A_1_ was recorded at 515 nm. The DPPH solution was swapped for absolute methanol, and the absorbance (A_2_) of this mixture was measured. The sample solution was substituted with distilled water, and absorbance A_0_ was measured. The scavenging percentage was determined via the following equation:

Scavenging rate = [1 − (A_1_ − A_2_)/A_0_] × 100%.

#### 4.11.2. Hydroxyl Radical Scavenging Activity

As noted in studies [49,50], 50 μL of the polysaccharide sample solution at various concentrations, prepared in distilled water, was mixed in a test tube with 100 μL each of 9 mmol/L ferrous sulfate and 9 mmol/L salicylic acid (prepared in anhydrous ethanol) in a well of a 96-well plate. Subsequently, 100 μL of 8.8 mmol/L hydrogen peroxide was introduced, and the resulting mixture underwent a 30 min reaction at 25 °C. Following a 5 min spin at 12,000× *g*, the 532 nm absorbance (A_1_) was quantified. The absorbance A_2_ was measured by replacing hydrogen peroxide with distilled water. To establish the baseline absorbance (A_0_), distilled water was used to replace the sample solution as a reference. The hydroxyl radical scavenging activity was then computed using the following equation:

Scavenging Rate = [1 − (A_1_ − A_2_)/A_0_] × 100%.

### 4.12. Tests Evaluating CPAP-1’s Protective Impact on HUVECs Subjected to H_2_O_2_-Induced Damage

Human umbilical vein endothelial cells (HUVECs) at peak growth were harvested and seeded into 96-well plates, using complete medium (DMEM + 10% FBS), with each well holding approximately 5000 cells. Following seeding, the cultures were incubated overnight at standard physiological conditions, 37 °C with 5% carbon dioxide, to allow proper cell attachment and acclimation. After incubation, the original medium was aspirated and replaced with a blank medium containing either 0.4 mol/L H_2_O_2_, different mass concentrations of CPAP-1 (0.0625, 0.1250, 0.2500 mg/mL), or their combinations, depending on the experimental group. Both H_2_O_2_ and CPAP-1 were dissolved and diluted in the blank medium. The experiment included a blank control group (blank medium only), a model group (treated with 0.4 mol/L H_2_O_2_ only), and intervention groups (treated with both 0.4 mol/L H_2_O_2_ and various concentrations of CPAP-1). Post 24 h incubation, CCK-8 (10 μL) was introduced to each well, and then an extra 4 h of incubation was conducted. The OD value was measured at 450 nm with an enzyme marker. Cell viability was then determined based on the prescribed calculation method. CPAP-1’s mitigation of H_2_O_2_-triggered HUVEC injury was gauged via the cell survival rate.

### 4.13. Statistical Analysis

All experiments were conducted in triplicate, and results were presented as mean ± standard deviation (SD). To determine statistical significance, a one-way analysis of variance (ANOVA) was conducted using SPSS 23.0, followed by the Least Significant Difference (LSD) post hoc test, with a *p*-value less than 0.05 denoting a significant variation.

## 5. Conclusions

This study successfully established a purification protocol for obtaining CPAP-1, a novel homogeneous polysaccharide from *Curcuma phaeocaulis*. Comprehensive structural analysis proposed that CPAP-1 possessed a unique arabinogalactan backbone primarily composed of →3,6)-*β*-d-Gal*p*-(1→ and →3)-*β*-d-Gal*p*-(1→ glycosidic linkages, a structural motif rarely reported in this plant source. Beyond its structural novelty, CPAP-1 exhibited remarkable antioxidant efficacy, demonstrating potent free radical scavenging capacity. More importantly, it effectively protected HUVECs from H_2_O_2_-induced oxidative damage at the cellular level, suggesting its potential mechanism may involve direct neutralization of reactive oxygen species and enhancement of cellular resistance. The antioxidant profile of CPAP-1 lends support to the view that the antioxidant activity of sugars may depend on a combination of factors, including chemical composition, structural characteristics, and component synergism, rather than any single element. These robust bioactivities confirmed its significant potential as a natural antioxidant. Our findings indicated that CPAP-1 represented a promising candidate for development as a functional food ingredient or a potential therapeutic agent for alleviating oxidative stress. Future work will pursue in vivo validation of antioxidant efficacy and elucidation of structure–activity relationships at the molecular level.

## Figures and Tables

**Table 1:** Methylation and GC–MS assessment of CPAP-1 connectivity.

Sugar Derivatives	Diagnostic Fragments (*m*/*z*)	Relative Molecular Weight	mol %	Deduced Residues
1,5-di-*O*-acetyl-6-deoxy-2,3,4-tri-*O*-methyl mannitol	59, 72, 89, 102, 115, 118, 131, 145, 162, 175	418	2.10%	t *-Rha(*p*)
1,4-di-*O*-acetyl-2,3,5-tri-*O*-methyl arabinitol	71, 87, 102, 118, 129, 145, 161	7678	38.47%	t-Ara(*f*)
1,2,4-tri-*O*-acetyl-3,5-di-*O*-methyl arabinitol	88, 101, 129, 130, 161, 190, 233	375	1.88%	2-Ara(*f*)
1,3,4-tri-*O*-acetyl-2,5-di-*O*-methyl arabinitol	87, 99, 113, 118, 129, 201, 233	1182	5.92%	3-Ara(*f*)
1,5-di-*O*-acetyl-2,3,4,6-tetra-*O*-methyl galactitol	87, 102, 118, 129, 145, 161, 162, 205	2269	11.37%	t-Gal(*p*)
1,4,5-tri-*O*-acetyl-2,3-di-*O*-methyl xylitol	87, 102, 118, 129, 162, 189	1004	5.03%	4-Xyl(*p*)
1,3,5-tri-*O*-acetyl-2,4,6-tri-*O*-methyl galactitol	87, 101, 118, 129, 161, 202, 234	1282	6.42%	3-Gal(*p*)
1,4,5-tri-*O*-acetyl-2,3,6-tri-*O*-methyl glucitol	87, 102, 113, 118, 129, 162, 233	1008	5.05%	4-Glc(*p*)-UA
1,2,4,5-tetra-*O*-acetyl-3-*O*-methyl arabinitol	87, 88, 129, 130, 189, 190	255	1.28%	2,5-Ara(*f*)
1,2,3,4,5-penta-*O*-acetyl arabinitol	85, 86, 103, 115, 116, 128, 145, 146, 159, 188, 201, 218, 290	756	3.79%	2,3,5-Ara(*f*)
1,3,5,6-tetra-*O*-acetyl-2,4-di-*O*-methyl galactitol	87, 101, 118, 129, 160, 189, 234	3733	18.70%	3,6-Gal(*p*)

* t: terminal.

**Table 2:** ^1^H- and ^13^C-NMR spectral parameters for key carbohydrate components in CPAP-1.

Code	Glycosyl Residues	Chemical Shifts (ppm)					
		H1/C1	H2/C2	H3/C3	H4/C4	H5/C5	H6/C6
Residue A	*α*-l-Ara*f*-(1→	5.19	4.16	3.89	4.08	3.68, 3.78	/
		109.22	81.32	76.57	83.84	61.26	/
Residue B	→3,6)-*β*-d-Gal*p*-(1→	4.48	3.6	3.7	4.09	3.89	3.87, 3.99
		103.14	69.87	80.09	68.45	73.38	69.62
Residue C	*β*-d-Gal*p*-(1→	4.41	3.49	3.62	3.89	3.66	3.79
		103.57	71.31	72.74	70.74	75.85	61.74
Residue D	→3)-*β*-d-Gal*p*-(1→	4.44	3.5	3.61	3.72	4.09	3.63
		102.63	71.77	80.36	70.02	75.52	61.01
Residue E	→3)-*α*-l-Ara*f*-(1→	5.03	4.11	3.89	4.09	3.63	/
		107.47	80.86	83.99	81.03	60.91	/
Residue F	→4)-*α*-d-Glc*p*A-(1→	4.98	3.78	4.08	n.d	n.d *	/
		99.55	68.87	68.15	n.d	n.d	175.68
Residue G	→4)-*β*-d-Xyl*p*-(1→	4.43	3.31	3.5	3.68	3.67, 3.77	/
		103.31	73.46	75.08	76.47	63.27	/
Residue H	*β*-l-Rha*p*-(1→	4.69	3.92	3.71	3.37	3.98	1.2
		100.68	68.61	70.47	72.00	n.d	16.54
Residue I	→2,3,5)-*α*-l-Ara*f*-(1→	5.39	4.24	4.13	4.05	3.76	/
		107.74	87.28	81.93	84.08	66.7	/
Residue J	→2,5)-*α*-l-Ara*f*-(1→	5.24	4.17	3.95	4.14	3.85	/
		108.42	82.24	76.95	81.96	68.92	/
Residue K	→2)-*α*-l-Ara*f*-(1→	5.13	4.01	3.86	4.19	3.69	/
		106.98	83.98	82.08	81.87	59.34	/

* n.d: not detected.

## Data Availability

The data presented in this study are available upon request from the corresponding author.

## Supplementary Materials

The following supporting information can be downloaded at https://www.mdpi.com/article/10.3390/molecules30224340/s1, Figure S1. Elution curve of CPP on DEAE seplife FF column; Figure S2. Elution curve of CPP on Sephacryl S-400 HR column; Figure S3. Original NMR spectra of CPAP-1. Table S1. Extraction rates and purities of each polysaccharide component.
