# Supplementary material for: Structural Characterization and Protective Effects of CPAP-1, an Arabinogalactan from Curcuma phaeocaulis Val., Against H2O2-Induced Oxidative Damage in HUVECs
Source: Molecules. 2025 Nov 9;30(22):4340. doi: 10.3390/molecules30224340 (PMC12655686; doi:10.3390/molecules30224340)
Supplement: Supplementary file 1 [file molecules-30-04340-s001.zip › molecules-3947905-supplementary.pdf]

# Structural characterization and protective effects of CPAP-1, an arabinogalactan from *Curcuma phaeocaulis* Val., against $H_2O_2$-induced oxidative damage in HUVECs

**Yuhao Long[1, 2], Sirui Yi[1 2], Huizhi Zhou[2], Fangrou Chen[3], Yiping Guo[1, 2 *], Li Guo[1, 2 *]**

1 *Key Laboratory of Standardization of Chinese Medicine (Chengdu University of Traditional Chinese Medicine). Ministry of Education, Chengdu 611137, Sichuan, China;* longyuhao@stu.cdutcm.edu.cn (Y.L.)

2 *School of Pharmacy, Chengdu University of Traditional Chinese Medicine, Chengdu 611137, Sichuan, China;* yisirui@stu.cdutcm.edu.cn (S.Y.); zhouhuizhi@stu.cdutcm.edu.cn (H.Z.)

3 *College of Clinical Medicine, Chengdu University of Traditional Chinese Medicine, Chengdu 611137, Sichuan, China;* chenfangrou@stu.cdutcm.edu.cn (F.C.)

* Correspondence: yguo8@ucmerced.edu (Y.G.); guoli@cdutcm.edu.cn (L.G.); Tel.: +86-138-8172-1018 (L.G.)

**Table captions**

Figure S1. Elution curve of CPP on DEAE seplife FF column.

Figure S2. Elution curve of CPP on Sephacryl S-400 HR column.

Figure S3. Original NMR spectra of CPAP-1.

Table S1. Extraction rates and purities of each polysaccharide component.

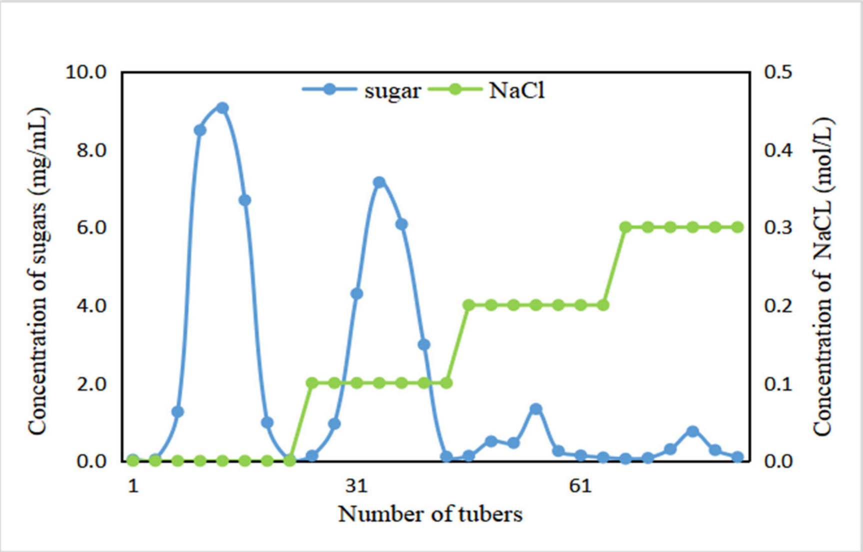


**Figure. S1.** Elution curve of CPP on DEAE seplife FF column.

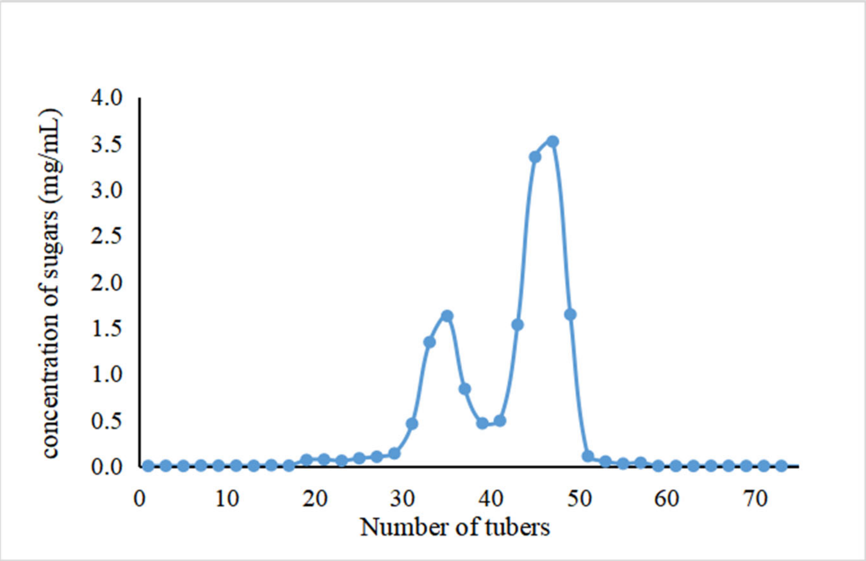


**Figure. S2.** Elution curve of CPAP on Sephacryl S-400 HR column.

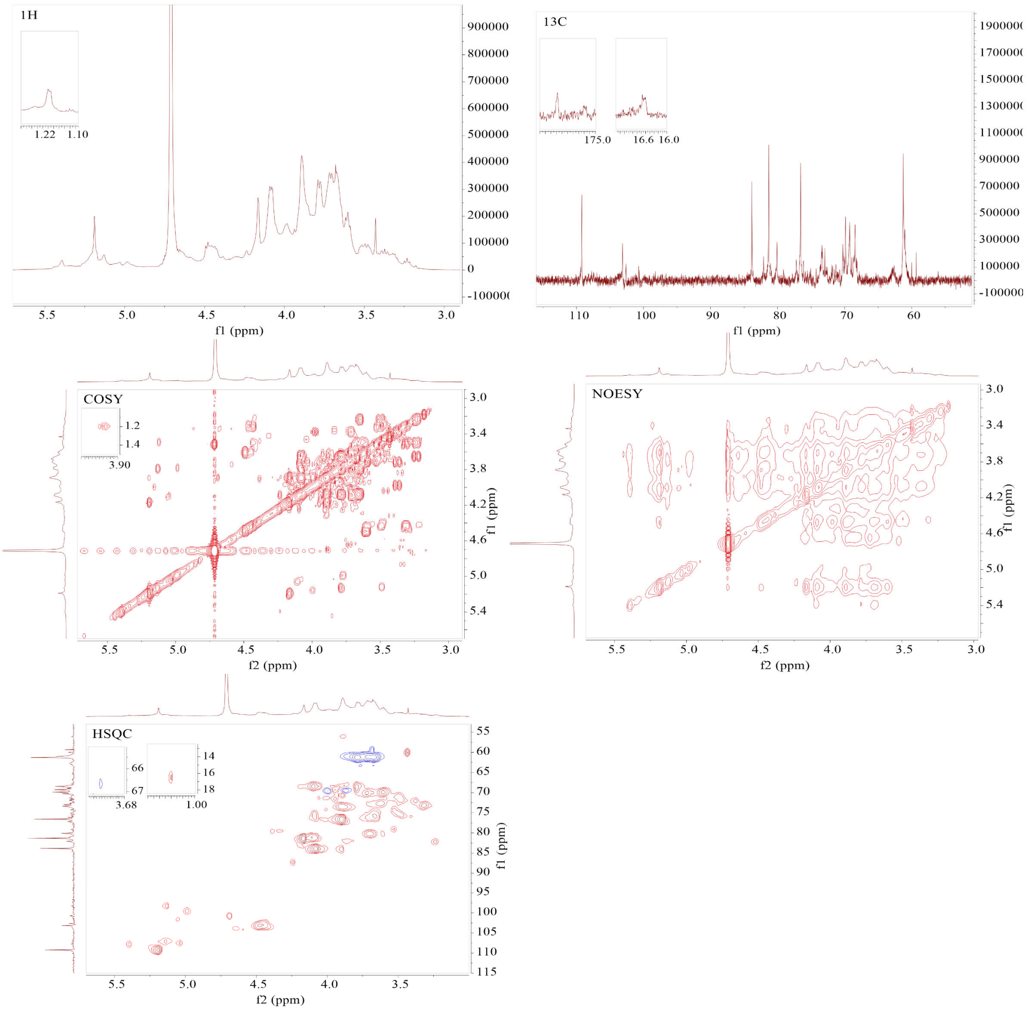


**Figure S3.** Original NMR spectra of CPAP-1.

**Table S1.** Extraction rates and purities of each polysaccharide component.

| Component | Extraction rate | Purity |
|---|---|---|
| CPP | 0.64% | 65.4% |
| CPNP | 20.8% | 67.5% |
| CPAP | 16.6% | 82.3% |
| CPAP-1 | 18.2% | 95.3% |
